# Psychiatric Assessment of a Non-Related Altruistic Living Kidney Donor: A Case Report

**DOI:** 10.1192/j.eurpsy.2026.11051

**Published:** 2026-07-31

**Authors:** B. Marşap, I. Ekmekci

**Affiliations:** Psychiatry, Gazi University Hospital, Ankara, Türkiye

## Abstract

**Introduction:**

Chronic kidney disease is associated with high morbidity and mortality, its gold standart treatment is renal transplantation. Unfortunately there is a shortage of cadaveric donors. Therefore living kidney donation rates are increasing, including altruistic donations to non-relatives. The psychiatric evaluation of living donors is crucial to ensure voluntariness, decisional capacity, and also to minimize post-donation psychiatric risks. However there is limited data regarding the psychiatric assessment and outcomes of altruistic donors in Türkiye.

**Objectives:**

Our objective is to present the psychiatric evaluation process of an altruistic kidney donor who was not a first-degree relative of the recipient. We also want to discuss challenges in assessing motivation, voluntariness, and psychological suitability for donation.

**Methods:**

A 53-year-old male altruistic kidney donor candidate was evaluated in a tertiary care university hospital and was referred for psychiatric consultation by the transplant team. The assessment included comprehensive psychiatric interviews and mental status examination, review of medical and psychiatric history, administration of MMPI and Rorschach tests, multiple interviews (total 9 sessions in 12 days) to assess consistency of motivation and emotional stability. The donor’s comprehension of surgical risks, postoperative course, and potential psychosocial impact was assessed. Donor’s consent, judgement, understanding of risks, and psychological suitability were confirmed by two psychiatrists and reviewed with the transplant team.

**Results:**

The donor was a 53-year-old single man with no children, living alone and working in a friend’s shop. He reported a close but not intimate relationship with the recipient. He he had known the recipient since childhood from the neighborhood. The donor’s judgement and insight was intact, his mood was euthymic. No active or history of psychotic or affective episode was detected. Although there was a past history of a depressive episode which he was prescribed psychotropics, the patient had no active psychiatric complaints and symptoms. Psychological testing indicated possible narcissistic and impulsive traits but no contraindication to donation was found. Motivation was consistent, altruistic, and free of coercion or expectation of secondary gain. The case was approved for donation, and the nephrectomy was successfully performed. Postoperatively, the donor reported stable mood and no psychological complications.

**Conclusions:**

This case highlights the importance of detailed psychiatric evaluation in altruistic non-relative kidney donation. The psychiatric evaluation includes repeated interviews and psychological testing to ensure voluntariness and rule out psychiatric contraindications. Multidisciplinary collaboration between psychiatry and transplant team and standardized guidelines are needed to ensure donor safety and ethical integrity.

**Disclosure of Interest:**

None Declared

